# Montreal Veterinary Medical Association

**Published:** 1896-12

**Authors:** B. A. Sugden

**Affiliations:** Secretary-Treasurer


Secretary.

At the meeting of the Niagara and Orleans County Veterinary Medical Society on the 9th of October, the further consideration of the adoption of a constitution and by-laws were considered, after which the meeting adjourned to convene at Niagara Falls in January, 1897.

### MONTREAL VETERINARY MEDICAL ASSOCIATION.

THE twenty-second annual opening meeting was held in the library of the Faculty of Comparative Medicine on Thursday evening, October 8th, the Hon. President, Dr. D. McEachran, occupying the chair. Dr. Charles McEachran was also present. Dr. McEachran, in his opening remarks, spoke of the great value of such societies to the student and to the profession in particular. He then resigned the chair in favor of the retiring President, Dr. Baker.

The business of the evening—viz., the election of officers—was then proceeded with. Dr. Baker was re-elected President and the following gentlemen to the various offices named: Dr. Mills, First Vice-President; Dr. C. McEachran, Second Vice-President; Dr. Thurston, Librarian; B. A. Sugden, Secretary and Treasurer. Eight new members were added to the roll. After other routine business had been completed the meeting adjourned.

The second meeting was held in the library, Thursday evening, October 22d, the President, Dr. M. C. Baker, occupying the chair. There was a good attendance of members.

The Secretary was instructed to obtain Metchnikoff's *Researches on Comparative Inflammation* and Dr. Clement's work on *Past-mortems* for addition to the library.

Mr. W. B. Wallis than reported a case of "Parturient Apoplexy in a Cow," rendered more interesting by the subsequent occurrence of subcutaneous emphysema, which he attributed to some traumatism of the lung-tissue, which accords with Zeigler's theory, in which he maintains that the condition may occur by the passage of air through a wound in the lung-tissue, and extension by way of the lymphatic and connective-tissue spaces to the subcutaneous tissues. In the discussion that followed some interesting facts as to the treatment of milk-fever were brought out. This case made a good recovery under strychnine and stimulant treatment. The President pointed out the ample field for research which this disease presented to students. Dr. Martin, in his remarks on emphysema, showed that in the human subject the condition was mainly of microbic origin, being due to certain air-forming bacilli.

Dr. Burns then read a paper on "Nursing and General Management of the Sick. "After touching on the important points in connection with this subject, such as pure air, scientific and rational methods of feeding, etc., he went on to show how seldom these fundamental principles were put into practical execution in veterinary medicine. At the close of his paper Dr. Burns illustrated by means of diagrams several plans of ventilation and drainage which he had personally observed in some of the large cities in the United States. One of these consisted of an air-shaft which ran all round the stable above the stalls and boxes, and over each stall and box were apertures in the shaft which could be opened and closed at will, according to the condition of the patient occupying the space below. Dr. Martin pointed out that a large proportion of veterinary cases were due to irritation of the gastro-intestinal tract, caused by irregular and improper feeding. As regards the training of attendants, the President laid great emphasis upon the value of precept and example, and, in giving the benefit of his experience concerning the temperature of stables, recommended one of from 50° to 55° during the winter months.

The essayist for the next meeting having been notified, the proceedings terminated.

A meeting was held in the library of the Faculty of Comparative Medicine, Thursday evening, November 5th, Dr. Mills occupying the chair, and Drs. D. McEachran, Baker, and Charles McEachran being also present.

There being no business the chairman called upon Mr. Hilliard to report his case. This was one of "Lithotomy," performed on an aged gelding, in which a calculus, loose in the bladder, had been diagnosed, causing frequent attempts at urination. Owing to the size of the obstruction, which proved to weigh 1700 grains (about the size of a hen's egg), the following operation was performed: An incision was made through the perineum down and into the urethra; but on bringing the obstruction to the neck of the bladder by means of the forceps and the hand introduced into the rectum, it was found to be too large for removal. An incision was therefore made in the neck of the bladder by means of a concealed knife, after which the calculus was withdrawn without difficulty. Slight hemorrhage followed the operation, but, an injection of cocaine having been given, the animal evinced but little pain, merely arching his back during the passage of the stone, and eventually made a good recovery.

Mr. Connelly followed with a paper on "Influenza," tracing its history from the time when it was recognized as an epizoötic among the war-horses of Rome, and later, in 1776, visiting America, where it has existed up to the present time. Horses about the age of four or five years are most liable to an attack, one of which is supposed to give immunity from others. A period of incubation of from five to seven days occurs before any symptoms present themselves, after which they may be so slight as to attract but little attention, but, on the other hand, are most frequently serious, and present the appearance of rapidly developing fever with marked depression, and when accompanied by grinding of the teeth a severe attack may be expected, the temperature occasionally rising to 107° F. in the first twelve hours. The conjunctiva is œdematous, and this, with excessive lachrymation, has given rise to the name of "pink-eye." The disease may terminate rapidly and favorably or complications of almost any organ may ensue. In the former case the duration does not exceed from four to eight days. Treatment: While the appetite remains the patient should have a moderate quantity of fresh clover, if it be obtainable, warm bran-mashes, boiled oats, or linseed meal. Clothe the body and extremities, and place the animal in a well-ventilated, loose box. Antipyretics are indicated. Bleeding is questionable treatment, and if ever employed it must be at the outset of the disease. Steaming the head is good practice, as it relieves the cough and nasal discharge. When much debilitated stimulants should be used, and in connection with a weak heart digitalis has proved advantageous. In the discussion which ensued the following interesting points were mentioned by Dr. D. McEachran—namely: That complications affecting certain organs were generally due to some previous disease of said organ having left it in a weakened condition and therefore predisposed to disease. In all cases there is cellulitis and thickening of the membrane, and the doctor mentioned that in his opinion the disease was doubtless of microbic origin, the specific germ of which he was sure would ere long be demonstrated. Dr. Baker pointed out that in differential diagnosis between an ordinary cold and influenza one should take into consideration that in the latter case great depression is present, and that a large number of animals are similarly affected without any apparent cause. He recommended as treatment the administration in the drinking-water of one drachm of nitrate of potash and one ounce of magnesium sulphate three times a day; as a sequel he described swelling principally of the flexor tendons accompanied by shifting or rheumatic lameness. Dr. Charles McEachran drew attention to the general occurrence of roaring as a result of influenza, it probably being due to an enlarged condition of the bronchial lymphatic glands interfering with the function of the left recurrent laryngeal nerve, and pointed out the importance of this circumstance as regards a certificate of soundness, which he recommended should not be given without modification during the first three months following influenza.

The proceedings then terminated. B. A. SUGDEN,
Secretary-Treasurer.

